# Updating our understanding of situation awareness in relation to remote operators of autonomous vehicles

**DOI:** 10.1186/s41235-021-00271-8

**Published:** 2021-02-19

**Authors:** Clare Mutzenich, Szonya Durant, Shaun Helman, Polly Dalton

**Affiliations:** 1 Royal Holloway, University of London, London, UK; 2 Transport Research Laboratory, Crowthorne, UK

## Abstract

The introduction of autonomous vehicles (AVs) could prevent many accidents attributable to human driver error. However, even entirely driverless vehicles will sometimes require remote human intervention. Current taxonomies of automated driving do not acknowledge the possibility of remote control of AVs or the challenges that are unique to such a driver in charge of a vehicle that they are not physically occupying. Yet there are significant differences between situation awareness (SA) in normal driving contexts and SA in these remote driving operations. We argue that the established understanding of automated driving requires updating to include the context of remote operation that is likely to come in to play at higher levels of automation. It is imperative to integrate the role of the remote operator within industry standard taxonomies, so that regulatory frameworks can be established with regards to the training required for remote operation, the necessary equipment and technology, and a comprehensive inventory of the use cases under which we could expect remote operation to be carried out. We emphasise the importance of designing control interfaces in a way that will maximise remote operator (RO) SA and we identify some principles for designing systems aimed at increasing an RO’s sense of embodiment in the AV that requires temporary control.

## Significance statement

Personal motorised mobility is central to modern life. Autonomous vehicles (AVs) offer a range of potential benefits to society and to individuals such as mobility solutions for those who cannot drive themselves in the form of ride-sharing or autonomous taxi services, and reducing the number of road collisions that stem from errors in human judgement. AVs also provide plausible solutions to the issue of overcrowded highways as connected cars will communicate with each other and navigate an effective route based on real-time traffic information, making better use of road space by spreading demand (Department for Transport 2015). The 'Waymo Driver' self-driving taxi service is operating in California and has already accumulated over 20 million miles on open roads (Waymo 2020). GM owned AV 'Cruise' received a permit from the California DMV in October 2020 to remove the human backup driver from their self-driving cars and their 'Origin’ prototype will have no steering wheel or pedals (California DMV 2020). This activity strongly suggests that the next few years will see a transition towards ever-increasing levels of vehicle autonomy. Yet the impression that driverless cars will mean there is no human involvement, since there is no human physically present in the vehicle, is a fundamental misconception (Cooke 2006). In reality, many problems can arise that would require a human operator to remotely assess and instrumentally correct or direct the automation as AVs are not able to perceive some information that humans take for granted (Adams 2007). An understanding of the challenges for remote operators of automated vehicles and considering them as a part of the automation process is thus an urgent research priority.

## Introduction

Driving is an integral part of many people’s lives—commuting to and from work, visiting friends or travelling across the country. The introduction of autonomous vehicles (AVs) could make more effective use of this time and prevent many accidents that are attributable to human driver error (Department for Transport 2014). However, even entirely driverless vehicles will sometimes require human intervention, which will often need to be provided remotely in the case of vehicles with no ‘backup’ driver present in the vehicle. This article, whilst not a formal systematic review, considers the extent to which our understanding of situation awareness requires updating to encompass these new contexts via a detailed examination of the current state of the art in remote operation of autonomous vehicles.

**Fig. 1:**
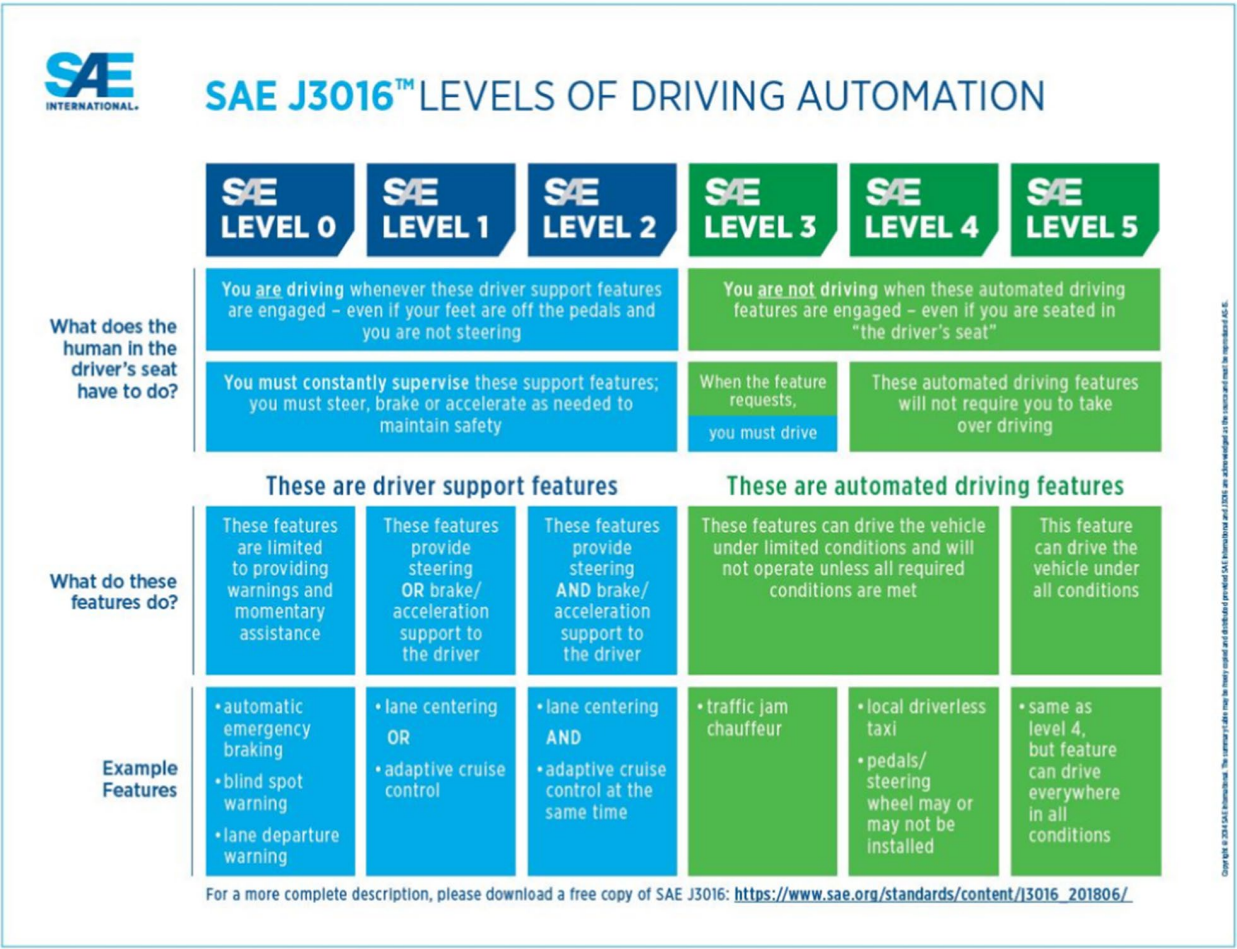
SAE International’s (2016) six levels of automation for on-road vehicles, providing a universal taxonomy of terms for describing and defining levels of automation

The organisational body SAE International (2016) highlighted six levels of automation for on-road vehicles (Fig. 1), with the aim of providing a universal taxonomy of terms for describing and defining levels of automation which can be adopted by industry, manufacturers and media. Levels 0–2 require the driver to be in charge at all times but with some partial assistance from enhanced or warning systems such as automatic braking systems. At Level 3 (conditional automation), the car can drive alone for short periods, merging onto motorways or changing lanes, however the driver is always physically present in the driver’s seat, ready to take over if the car requests intervention. This assumes that the human is monitoring the driving environment either implicitly or explicitly and will be able to quickly re-engage with the driving process (Gugerty 1997, 2011). Tesla's model S offers a 'fully self-driving' mode which is, in fact, a Level 3 system as the driver is required to take over, meaning they must be in the driving seat and ready to respond in a timely fashion to takeover requests (despite high profile videos showing drivers sitting in the passenger or back seat (Mills 2018).

At Level 4 (high automation), the car can handle all dynamic driving tasks and should not require the human to take over driving. However at this level the AV is limited to its Operational Design Domain (ODD) and the car is programmed to achieve a minimal risk condition (MRC) by coming to a safe stop if there is a problem (Pollard 2018). The ODD may be controlled areas such as geofenced metropolitan zones or motorways, or may refer to a strictly defined route or be determined by weather conditions, speeds or time of day (Wood et al 2019). Level 4 AVs are likely to be autonomous shuttles which operate in small precincts or districts with a limited route and low speeds (less than 25 mph) such as the driverless shuttle trials offered by Oxbotica at Gatwick Airport (Oxbotica 2020).

By Level 5 (full automation), the passenger is required only to set the destination and start the car, as the automated driving system can operate full time performance of the driving brief. Levels 4 and 5 are differentiated by the fact that at Level 5 the vehicle is not restricted to an ODD and can operate on any road where a human could drive a car (SAE International 2018). The Automated and Electric Vehicles Act 2018 defines a vehicle as “driving itself” if it is operating in a mode where it is not controlled or monitored by a human (Law Commission, 2018, p. 9). Level 5 is seen as the final stage of automation where the car is fully self-driving and the human occupant would never be required to take over the driving.

Although there may be long periods of self-driving in a Level 4 or 5 AV, it seems disingenuous to expect zero system failure. This fact is widely recognised, with some industry experts, such as Waymo's CEO, even claiming that Level 5 autonomation is not achievable given the wide range of technical challenges involved in driving in "all conditions" (SAE International 2018, p. 2; Tibken 2018). The belief that humans can be finally "automated out of the loop" still proves to be a fundamental misconception, illustrating years of overconfidence in technology (Cooke 2006, p. 166). Problems will inevitably arise that are beyond the capability of the AVs’ programming, obliging human involvement in the loop to assess the situation and instrumentally correct or direct the automation.

Until 2020, for AVs to be tested on public roads, legislation universally required that a safety driver must be inside the vehicle, at the wheel ready to take over if a disengagement was requested. However, changes to many European, US state and UK regulations have enabled a remote operator to assume this role. The Californian Department of Motor Vehicles (DMV) (the state that has the highest number of registered companies testing AVs on public highways) defines a remote driver as one not required to sit in the driver's seat. They may be able to monitor and communicate with the AV and may be able to perform driving or cause the AV to assume the MRC) which is usually coming to a safe stop (California DMV 2018). The United Nations Economic Commission for Europe (UNECE) considers remote operation as a key priority for regulation and has called for the definition of automated driving to be broadened to include remote support (UNECE 2020). In the UK in 2020, the Centre for Connected Vehicles (CCAV) regulated by the British Standards Institute (BSI), published two revisions to previous legislation, permitting remote operation to bring the AV to a controlled stop. The SAE Recommended Practice J3016 recognises and outlines the role of a remote driver as,

a driver who is not seated in a position to manually exercise in-vehicle braking, accelerating, steering, and transmission gear selection input devices (if any) but is able to operate the vehicle. (SAE International 2018, pg. 16).

Furthermore, The Law Commission (2018) proposes that all AVs should have a person present who is able to take over the driving of the car if required, not a driver but a ‘user in charge’ who may be inside or outside the vehicle.

The handover from an AV to a safety trained human operator is referred to as a disengagement (Khattak et al. 2020). We can study data from disengagements to consider how frequently human operators may be required to re-join the loop. Currently, only the state of California records how many disengagements each company has per number of miles driven in that year and records the reasons for the disengagement, for example perception or planning discrepancy and further details such as weather conditions and location of disengagement. In 2019, Waymo, the self-driving AV of Google-owned company Alphabet, drove the highest number of miles (1.45 million miles) and recorded 110 disengagements (one per 13,182 miles). Sixty-one of these were related to AV perception issues for example, "failure to detect an object correctly", "incorrect behavior prediction of other traffic participants" and "adverse weather conditions" (California DMV 2019). Further examples of reasons for disengagements by all companies included poor traffic light perception (Phantom AI), sun glare (Mercedes-Benz), construction zones (Valeo), small debris in the road (WeRide.com) and "too big rain" (Nullmax). These types of programming deficits are known in the automation business as edge cases (Davies 2018).

## Edge cases in autonomous vehicles

Edge cases vary significantly but they are colloquially defined as 'unknowable unknowns'—unpredictable, novel occurrences which fall outside the parameters defined by classifiers to recognise perceptual information (Koopman and Wagner 2017). They are triggered when an AV cannot correctly match the perceived information with known datasets, due to the presentation of ambiguous or incomplete stimuli. The neural networks that AVs rely on are trained on millions of images to enable the correct recognition and identification of a stimulus, however because edge cases are so unusual there are limited opportunities to train the system to recognise them (Hillman and Capaldi 2020). Gaps also exist in the datasets if an insufficient range of images have been used to train the algorithm, for example pedestrians may be labelled as walking on legs if disabled pedestrians were not shown in the training process, thus excluding wheelchair users (Koopman and Wagner 2016).

**Fig. 2:**
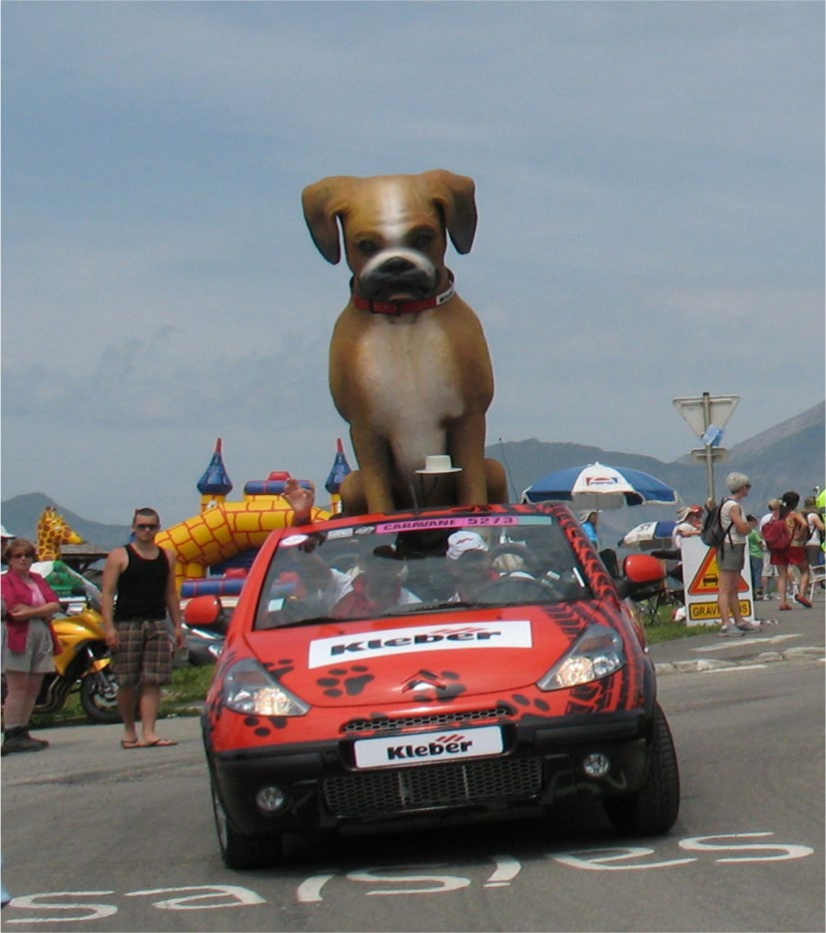
An example of an edge case; a large model of a dog travelling on a car may be classified as an animal, but its behaviour (travelling at speed) may not correspond with system expectations. This Photo by Unknown Author is licensed under CC BY-SA

**Fig. 3:**
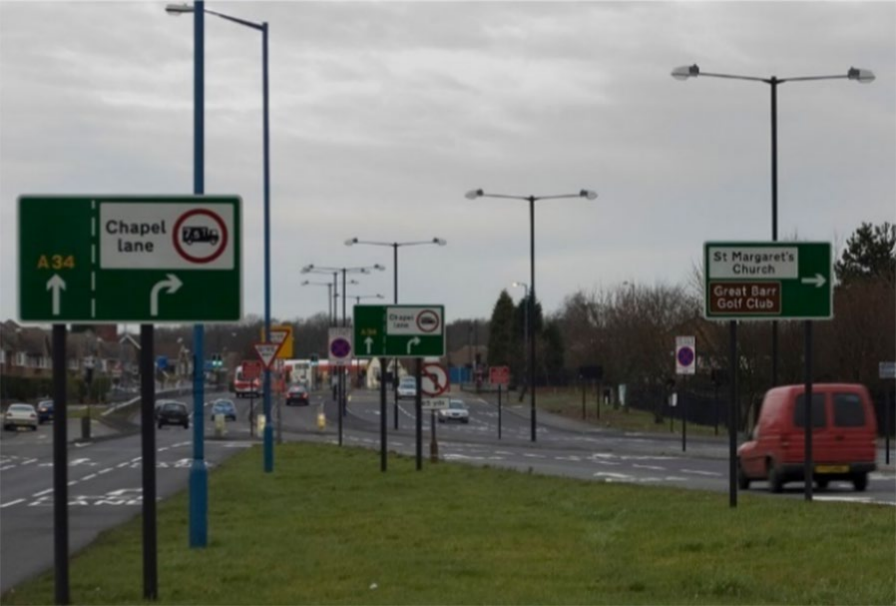
An example of edge case related to multiple signs. An autonomous system may interpret all visible signs as relevant, even though some relate to a parallel road. "Furniture" by hartlandmartin is licensed with CC BY-NC-SA 2.0

Edge cases can relate to animals, vehicles or objects presenting in unusual ways, for example a novelty trailer in the shape of a dog (Fig. 2) may be classified as an animal but its behaviour (travelling at speed on a motorway) may not correspond with system expectations which could trigger a minimal risk manoeuvre (MRM) such as an emergency stop. Further edge case examples may be generated by perception discrepancies caused by the target stimuli being occluded by other objects, such as a pedestrian moving behind a parked vehicle (California DMV Disengagement Reports 2019). Yet another example relates to the perception and interpretation of signage. The driving environment is frequently crowded with many signs cluttering both sides of the road offering competing information sources or even conflicting information. The Move_UK project found that some road signs only had a probability of detection of below 80%, mainly due to their location or angle, or whether they were obscured by vegetation or street furniture (Move_UK 2018). Human drivers are typically able to identify the relevant sign to their intended goal or destination and unconsciously filter out the other information, but an autonomous system may interpret all of them as relevant, particularly when it may not be apparent that they relate to a parallel road such as in Fig. 3. There is also evidence of consistent, systemic problems within many AV perception software systems to correctly 'see' or interpret cases of bare legs, children or red objects which raise significant safety concerns for pedestrians, vulnerable road users and traffic light adherence (Koopman 2020).

One approach to dealing with edge cases is to use human input in advance to teach an AV how a human would react in an emergency, but which can be applied by the AV in rapid time frames. The time it takes for an AV to assess the edge case could be reduced, meaning that time critical crashes could also be avoided, through the use of crowd sourcing, AI coordinated performance and reinforcement learning algorithms (Daw et al. 2019). The human is still required in this example to provide the human interpretation that the AV lacks, but they do so in advance, responding to randomly generated scenarios using simulation software which create a bank of potential actions that can be referenced by the AV. AVs predict risks frame by frame using vision-based software and if they see an unacceptable degree of perceptual uncertainty ahead they can access a library of suitable responses, eliminating the need to get in contact with an RO in that instance (Daw et al. 2019; Hampshire et al. 2020). It has been claimed that this type of assistance could have prevented the 2018 Uber crash in Arizona in which the system took 6 s to recognise the pedestrian pushing a bike as it did not conform to its expectations of cyclists or pedestrians, only determining 1.3 s before the collision that emergency braking was required (Daw et al. 2019; NTSB 2018). Even though this may forestall some edge cases relating to identification of stimuli, there are still many occasions where a human would need to remotely intervene to interpret the situation and determine the best course of action.

The likelihood of an AV encountering one of these edge case events grows with each passing mile, with the 676 autonomous vehicles registered in California alone in 2019 driving a total of 2,880,612 miles in 2019 (California DMV 2019). Even if an edge case was only encountered 0.01% of the time, that still represents a potential episode every 288 miles, although the technical software capabilities of AV companies vary significantly. Growing numbers of businesses offer services to stress test AV systems to find the edge cases in their software by assessing the "what-ifs", weaknesses and false negatives in the system, so that AV designers can mitigate those risks (www.edge-case-research.com). Developers can also use simulation software to focus on difficult driving scenarios and reproduce edge case scenarios, however these are still limited to human imagination and accordingly, in the words of Elon Musk, Tesla CEO, "simulation….does not capture the long tail of weird things that happen in the real world” (Wevolver 2020, pg. 40). Governments in the UK and the US have provided funding to research realistic edge case scenarios in simulated and real-world data. For example the D-risk project, part of a consortium of five organisations led by US start up, aiPod Limited, was awarded a £3 m grant in 2018 to use AI to develop a novel scenario generator by combining actual edge case scenarios (Centre for Connected & Autonomous Vehicles 2018). All this action illustrates the seriousness with which the industry is considering the impact of edge cases on Level 4 and 5 AVs.

Despite the continuing efforts to improve perception software in AVs, it is still likely that even if the AV is programmed to assume the MRC within its ODD and come to a safe stop, the car may still represent a risk to other road users or will need to be delivered to its destination. Indeed, there are still arguments as to whether the ODD at Level 5 is truly unlimited, as it may be unable to handle extreme weather conditions if its sensors fail (SAE J3016, 2016). Although a human may also struggle in some edge case scenarios, we possess the higher-level skills to interpret and react to novel scenarios. A scenario which represents an edge case for an AV may be easily interpretable by a human driver, suggesting that current automation technology still necessitates collaboration between humans and AVs (Hancock et al. 2019).

In a Level 3 (conditional automation) AV it is possible to take control by grabbing the wheel or hitting the brake. However, the future design of many Level 4 and all Level 5 vehicles could possibly have no steering control or brake at all. For example, the U.S. National Highway Traffic Safety Administration has recently approved the design and use of cars without steering controls and GM Cruise's new model, 'Origin', has no cockpit at all (Hawkins 2020; NHTSA 2016). In these types of AV, a human occupant would be unable to operate the car even if they wished to do so and so may need to call upon the services of a remote operator. Furthermore, there is disagreement amongst industry professionals as to whether the failsafe of performing the MRC in a Level 4 AV if the occupant cannot take over is an appropriate policy if the AV comes to halt in a line of traffic (Thorn et al. 2018). This may not in all cases represent a good strategy for the vehicle, its passengers or other road users, obliging some type of intervention by a remote operator who may be able to move the car to a more suitable location.

The functional scope that a remote operator offers can span from merely advising the AV of how to proceed, to interaction with passengers or to the extent of taking over the driving of the AV from line of sight or a remote location. The next section comprises an examination of the use cases under which we could expect a remote operator (RO) to be utilised and the roles or tasks the position may entail.

## Roles and use cases of a remote operator

**Fig. 4:**
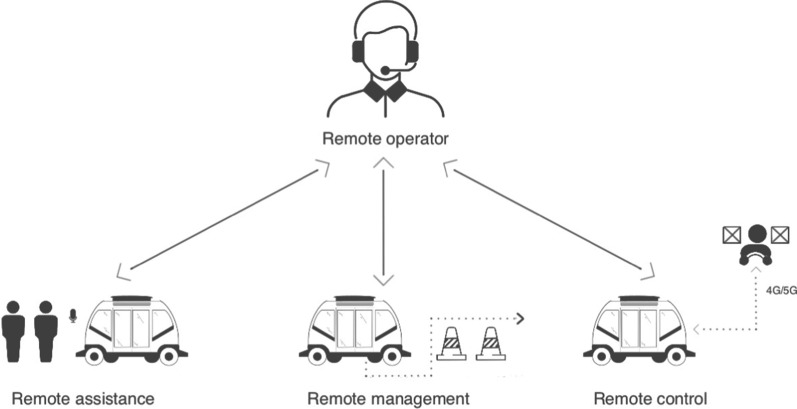
Visualisation of the roles a remote operator may provide to an AV in the event of an edge case

The three roles that a remote operator may be called upon to provide for an AV in the event of an edge case can be seen in Fig. 4. A remote operator (RO) may be required to provide **remote assistance** to a Level 4 and Level 5 AV by alerting the service provider when it has broken down or providing information and customer service to passengers (UNECE, 2020). For example, if the vehicle has a flat tire, a breakdown vehicle may need to be called and updates communicated to passenger as to how long the repair will take, whether an alternative vehicle will be dispatched etc. This type of service is already offered by GM's OnStar Crisis Assist program and AV companies such as AnyConnect and Sensible4 currently deploy remote control centres that are equipped to respond to customer service requests such as if a passenger demands to speak with an operator (Cummings et al. 2020, Sensible4.fi, AnyConnect.com). An RO may also be required to be responsible for the safety of passengers in self driving ride share situations, where there may be no conductor and the behaviour of other passengers may be a personal security risk (although this creates problematic surveillance and privacy issues for passengers on board which may need to be addressed by an on-call button rather than continuous audio monitoring). Even the simple task of opening and closing doors could be carried out by an RO or responding to a passenger request for an emergency stop (UNECE 2020).

A further role that could be offered by a RO is that of **remote management**, similar to an air traffic controller, where an RO working in a remote management post could also assume control of a fleet of AVs, as it would be poor economics to operate on a 1:1 basis (Hampshire et al. 2020). Fleet operations could include dispatch services which coordinate deliveries, navigational support and monitoring of radio and traffic information, for example communicating recent road closures to the entire fleet, as connected cars may wrongly interpret the low traffic volume as indicating the fastest route (Cummings et al. 2020). Giving the remote controller governance to order the AV to move or deviate from a fixed path would also enable the AV to override highway rules in exceptional circumstances, for example if instructed to do so by a police officer (UNECE 2020). Allowing driverless cars to call upon a centralised remote call centre means that several cars a day could be assisted using a blend of human experience and machine execution (Daw et al. 2019).

Furthermore, an RO may only need to assist in a purely advisory capacity in the event of an edge case triggering the MRC in a Level 4 or 5 AV (UNECE 2020). The RO could review the reason for the MRC and, after assessing the environment using the AV's cameras and sensors may confirm it is safe to proceed. Zoox is currently adopting this remote management strategy using 24 h support centres in which ROs provide human guidance to the AV, still in autonomous mode, in response to ambiguous scenarios (Zoox.com). The Texas A&M University shuttle program has introduced a self-driving trolley in the downtown precinct of Bryan, Texas, which is remotely monitored from a call centre where a RO authorises a restart when the shuttles is forced to stop for a pedestrian or object in the road (Costlow 2019).

This type of goal-based supervision could also be delivered in the form of a set of instructions e.g. for the AV to remove itself from the line of traffic where it may have assumed a MRC. It represents a prompt resolution to a vehicle obstructing traffic, but only requires basic training and carries less risk of human error than directly assuming control (Cummings et al. 2020). Nissan has also approached the challenge posed by edge cases by integrating remote management into its AV business model with its strategy of Seamless Autonomous Mobility. Operators working in a call centre, who Nissan refers to as Mobility Managers, plot a path for the AV around the object using a drawing interface and then return control to the AV to execute the path (Daw et al. 2019). The AV then relays the information to all the connected cars in its system, so each AV has that template to refer to in future similar situations, eventually reducing the need for an RO.

Lastly, the full test of an RO’s capabilities would be assuming sole responsibility for the dynamic driving task either at low or high speeds from a remote location separate to the physical environment where the AV is located (UNECE 2020). This type of **remote control** is referred to as teleoperation and could be a viable solution to expand the ODD of Level 4 and 5 AVs (Thorn et al. 2018). Teleoperation is a substitute or enhancement to driverless transport, using 4G or 5G mobile networks to continuously video stream visual data from cameras around the car and linking driving operations to a driving rig in a remote control centre via an on board unit in the car (T Systems 2020). The remote 'driver' receives real time information displayed on multiple monitors or through a VR headset and can control all acceleration, deceleration, steering and braking using a traditional steering wheel and pedals, or joystick (UNECE 2020).

Many companies are already offering teleoperation as a mobility solution for autonomous services: Ottopia supplies teleoperation capability for both indirect control (such as remote management already discussed) and direct control of AVs, partnering with fleet operation provider, Bestmile; Phantom Auto have employed their teleoperation software to remotely control unmanned vehicles, delivery bots and forklift trucks since 2017 in Mountain View, California; and WeRide in China have removed the safety driver and are insistent that remote driving is the next step in making AVs profitable (Dai 2019). Additionally, there were an increasing number of start-ups registered in 2019 that included remote teleoperation in their business model such as Scotty Labs, who partnered with Voyage supplying self-driving cars in retirement communities (Dai 2019). Six states in the US expressly allow for teleoperation and England, Canada, Japan, Finland and the Netherlands have also authorised its use in supporting autonomous vehicles.

However, there is intense debate within the automation industry as to what extent teleoperation as a service is a viable option, with companies such as TuSimple and 2GetThere rejecting it as an inherently unsafe prospect, others, such as Nissan, who consider an RO as a necessary precaution to edge cases, but do not include direct control, or others like Zoox and Einride who are factoring in remote operation of the AV but currently only in some instances/locations. There are also key differences between current teleoperation practices as to whether remote driving is delivered only at low speeds (less than 25mph) such as EasyMile electric shuttles or at high driving speeds such as Designated Driver who successfully remotely operated a car at Goodwood Racecourse (Cummings et al. 2020; Designated Driver 2019). It seems probable though, in the future, that some or all forms of remote operator will become an important feature to support autonomous driving. Thus, we need to reflect on the safety measures, performance requirements and the key issues that will be relevant to operators of remote vehicles.

The current article addresses two main issues with regard to the remote operation of an AV. Firstly, SAE International’s (2016) taxonomy does not acknowledge the possibility of remote handovers so suggestions are made to update the nomenclature. Secondly, ROs will face significant challenges that are unique to their role as driver in charge of a vehicle that they are not physically occupying. ROs are likely to require longer exposure times to gain sufficient situation awareness; they face latency and perception issues and may have difficulty achieving a sense of embodiment in the remote vehicle. We reflect on these issues and offer practical design features which may enable the RO role to be carried out within a safe and realistic framework.

## An extended taxonomy of automated driving systems

As described earlier, the SAE International levels of automation are adopted by all manufacturers to synchronise the classification of AVs and describe their capabilities at each level (SAE International 2018). We argue, however, that the SAE taxonomy does not reflect the fact that ROs will occasionally be obliged to intervene in the driving of an AV, for instance in the event of an edge case as previously discussed. The expectation has always been that the SAE taxonomy will change as the industry itself evolves, so we submit that the taxonomy now needs to be extended to include remote intervention by a remote operator who is effectively part of the AV system.

**Fig. 5:**
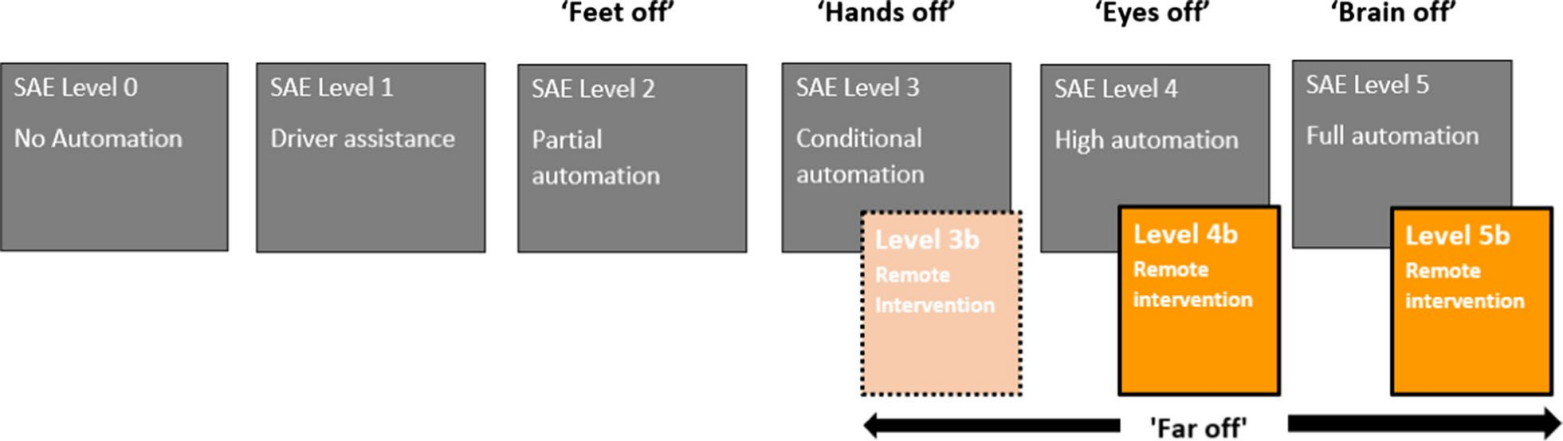
Suggested revisions to the SAE (2016) taxonomy to allow for the potential handover to an RO by the addition of Levels 3b, 4b and 5b to the existing Levels 3, 4 and 5. The extra level proposed could be summarised as 'far off', as the RO is intervening from a separate location (nb. ‘feet off, hands off, eyes off, brain off’ summary taken from Kemp 2018, p. 7)

We propose a revision to the SAE taxonomy to allow for the potential handover to an RO by the addition of Levels 3b, 4b and 5b to the existing Levels 3, 4 and 5 (see Fig. 5).

These adjunct levels encompass the three roles of the RO that we have outlined above (i.e. assistance, management and control), which are labelled collectively as ‘remote intervention’. The levels of automation have been informally reduced to ‘*feet off'* [Levels 0–2] as the car can take control of the pedals, *hands off* [Level 3] as the driver does not have to touch the steering controls, *eyes off* [Level 4] for when the driver no longer has to watch the road and '*brain off'* [Level 5] as the occupant could even fall asleep as the AV would never require them to take over (Kemp 2018, p. 7). The extra level that we propose could be summarised as '*far off'*, as the RO is intervening from a separate location possibly hundreds of miles away.

Remote intervention could potentially occur in the future even at Level 3 (indicated by the dotted lines and paler shading in Fig. 5) which is sometimes referred to as the ‘mushy middle’ of automation due to the AV’s capability to drive itself but with an occasional need for human assistance. Furthermore, at Levels 4 and 5, where there is no human ‘fail safe’ within the vehicle, edge cases should be expected to be more frequent, indicated in Fig. 5 by the solid line for Levels 4b and 5b.

**Fig. 6:**
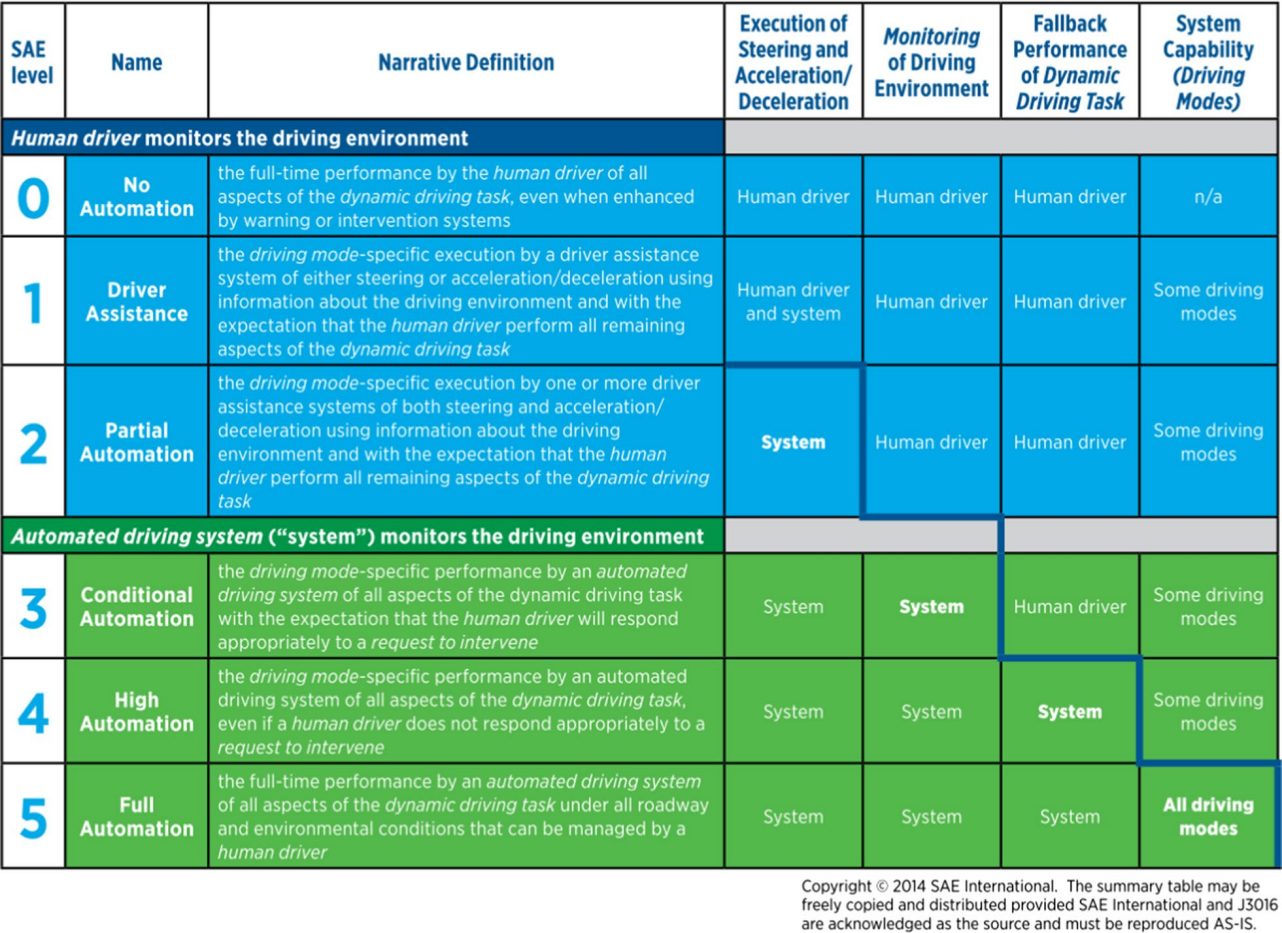
SAE International's (2014) summary of the responsibilities of human driver and system at each level of automation for the execution of steering and acceleration/deceleration, monitoring of the driving environment and fallback performance of the dynamic driving task

These additions represent a more comprehensive transition between system and remote human than the current SAE taxonomy for Levels 4 to 5 reflects, as it implies that the system **alone** is carrying out the execution of steering and acceleration/deceleration, monitoring of the driving environment and fallback performance of the dynamic driving task as is shown in Fig. 6. Instead, at Levels 4 and 5, the taxonomy should acknowledge the possibility of a reciprocal handover between system and remote operator, thus providing official recognition that edge cases will necessitate human–robot interaction for some time to come.

It is imperative to integrate the role of the remote operator within industry standard taxonomies so that regulatory frameworks can be established with regards to the training required for remote operation, the necessary equipment and technology, and a comprehensive inventory of the use cases under which we could expect remote driving to be carried out. An understanding of the unique challenges that remote operators of autonomous vehicles will encounter is subsequently an urgent research priority. We discuss these issues in the next section.

## Situation awareness in driving contexts

There are many definitions of SA (see Endsley et al. 2003; Gugerty 1997, 2011; Lo et al 2016; Niklasson et al. 2007, Endsley 2015) but the most commonly cited is from Endsley’s original model;

the perception of the elements in the environment within a volume of time and space, the comprehension of their meaning and the projection of their status in the near future (Endsley 1988a, b, p. 792).

Put more simply, SA fills the gap between what is known about the environment, what is happening in it and what might change. Endsley further divided the mechanisms of SA into three levels of responsiveness; Level 1, ‘Perception’ is the basic level of awareness that makes up the recognition of cues in the environment. Level 2, ‘Comprehension’, requires the current situation to be analysed, taking into account multiple pieces of information and their relative value to each other to make sense of what we are seeing. Level 3, ‘Projection’, a serial product of Levels 1 and 2, is the ability of the operator to make predictions about the future status of objects in their environment.

Endsley’s SA model is well-established in numerous domains and has proved to be applicable to driving contexts (Bolstad et al. 2010; Endsley 2019; Ma and Kaber 2005). Indeed, inadequate SA is frequently implicated in crashes; failed to look/distraction, a Level 1 SA error, is the most common citation in insurance documentation (Department for Transport 2014). The SA perception requirements for Level 1 for driving would include being aware of the *presence and location* of nearby objects such as pedestrians, other vehicles and objects and/or road signs, what time of day or night it is, and current weather conditions that may be hazardous (Endsley 2020). Awareness of the *distance* to nearby objects, vehicles in the blind spot and the traffic lane needed takes the SA state up to Level 2 (Comprehension) together with the *impact* of the weather and road conditions on vehicle safety (Endsley 2020). Perception and comprehension awareness are constantly updated during the driving process as the environment is dynamic and both the actions of the driver and other road users will affect the ongoing analysis of the situation. Driving also necessitates SA Level 3 projection of the likelihood of *collision* with other objects or vehicles, and estimated *times and distances* to turns or exits (Endsley 2020).

The three levels of SA can be mapped on to how an AV views and interprets the driving environment. For Level 1, in AVs, the automated system ‘perceives’ via sensors such as LIDAR, RADAR and multiple cameras which can see ‘through’ walls and under the surface of the road, although limited to visual and auditory inputs (Kemp 2018). However, the AV sensors can be fooled, and false positives can lead to emergency braking manoeuvres. For example, in the first phase of the Move-UK project, the AV mistook a cloud of exhaust smoke hovering over the street as a solid object and instructed the vehicle to stop, showing that human intervention may be necessary even at the level of simple perceptual judgements as they may require a degree of synchronised comprehension (Seidl 2018).

In terms of Level 2, ‘Comprehension’, AVs do not currently possess the level of artificial intelligence necessary to achieve the nuanced comprehension of humans. Many edge cases are context dependent and an AV may fail to detect details that a human would know are either important or irrelevant (Koopman and Wagner 2016). For example, a human driver may edge forward slowly through a crowd of pedestrians blocking the road to exert their right of way but would know that this behaviour was not appropriate if the crowd was surrounding a casualty on the road. This contextual distinction may not be as plain to a self-driving car so the unusual crowd behaviour would trigger a disengagement due to "interference of autonomous driving path by a pedestrian" (California DMV 2019; Law Commission 2018). This is when an RO could be required to take over from the AV in line with the remote management role outlined earlier to fill the “awareness gap” and either allow the AV to take a different path circumventing the crowd or instruct it to continue with the MRC (Drury et al. 2006, p. 91).

For Level 3, ‘Projection’, AVs currently struggle to make predictions with certainty as real-world driving is unpredictable and requires proactive as well as reactive decisions to avoid hazardous situations from developing (Endsley 2020). Humans are also unable to see into the future with certainty but we are capable of some anticipation, and of acting quickly and imaginatively in response to even unanticipated events; until AV software can demonstrate projection abilities at the same or at a greater level to those of a human driver, the requirement for remote operation is likely to remain, since an edge case is likely to still occur.

BSI guidelines in the UK, mentioned earlier, stipulate that the RO must be as safe, with the same level of situation awareness and response time, as a human safety driver assuming manual control of the car in the ODD (BSI 2020a, b). Research has attempted to quantify how much time it takes for drivers to build up SA in non-automated driving. Humans are capable of visually processing a natural scene within milliseconds as we can quickly pick up the gist of the contents (Thorpe et al. 1996). Lu et al. (2017) played participants videos of varying lengths and asked them to reproduce the traffic layout they had seen of the three-lane road. They found between 7 and 20 s was necessary to build up the necessary SA to complete this perceptual task successfully. However, when required to assess the relative speeds of other cars in relation to the ego vehicle, participants took 20 s or more. Fisher et al. (2007) found that the time it takes for drivers to become aware of latent or impending hazards in a scene was around 8 s and, in simulated scenarios, participants take around 12 s to feel safe enough to take over manual control of driving (Coster 2015). This suggests that perception of the world around you occurs quickly (Level 1 SA) but an understanding of what others are doing in that environment is slower (Level 2 SA) (Endsley 2017a, b).

## The need for a new understanding of remote SA

An RO who has been alerted by an AV to drop in and assess an edge case or assume direct driving control will first need to acquire SA of the remote scene, yet there are significant variations between SA in normal driving contexts as we have outlined above and SA as it might occur in remote driving operations. The task of developing SA from a remote location is likely to be made more difficult by the operator’s physical absence, however ROs will also have access to additional information (for example, from the advanced sensors on the vehicle in question, and from the sharing of information across entire fleets) such that some aspects of their SA will be enhanced by comparison to traditional driving. We accept Endsley’s Levels 1/2/3 as the core basis of how SA is constructed but argue in this article that a new consideration is needed in order to encompass the scenario of a remote ‘drop in’ to an AV.

Endsley’s model is most often considered as the operator, be it a military pilot or a road vehicle driver, being in-situ and experiencing information first-hand. In contrast, an RO is likely to suffer from a degraded state of SA as they are being transmitted indirect cues unlikely to replicate the full range of visual, vestibular, auditory and temporal information that is available to a driver in situ. Endsley clarifies her position by stating that SA is “the internal model of the world around […] at any point in time” (Endsley 1988a, b, p. 789). However, this cannot logically apply to an RO, as the world around them will be very different to that which they are experiencing though the video feed of the AV. For example, a forward camera view has reduced motion parallax information, which, together with a reduction in image quality, will reduce the depth cues available to a remote viewer, by comparison with someone situated within the scene itself. Similarly, the audio relayed to the RO from the scene (if any) will be of reduced quality and presented against the background noise of wherever the RO is physically present. However, on the other hand, the scope of cameras and other sensory information provided by an AV may give an RO superior awareness of some aspects of the environment, highlighting information that would be easy to neglect in person or is beyond the visual capability of humans.

Furthermore, Endsley (1988b, pg. 3) describes SA as "an *ongoing* process achieved by accumulating knowledge over the course of the mission" combined with the feedback from the *immediate* environment [our italics]. This will not be the case for remote operation of Level 5 cars as it is not feasible or efficient to monitor all cars on all roads constantly on a 1:1 basis. Instead, the most likely scenario would be a centralised control hub that AVs can contact when they encounter an edge case, which function using systems analogous to air traffic controllers; supplying remote management but also potentially delivering real-time remote operation and even 'look ahead' models which draw on human and artificial intelligence simulated interactions to cache pre-determined responses to potential situations (Daw et al. 2019). This type of teleoperation safety service can assist several cars a day and is already being offered in different forms by Silicon Valley start-ups, such as Phantom Auto, and autonomous trucking companies across Europe, such as Einride (Davies 2018).

Accordingly, although an RO will undoubtedly need to build up SA, their SA is not *ongoing*. An RO will ‘drop in’ to a scene having no prior exposure, meaning that they will need to develop SA from scratch, without access to previously accumulated knowledge for a particular vehicle and context. Neither does the RO occupy the ‘immediate’ environment that the car does. The likelihood of ROs having to unexpectedly take control of AVs in a wide variety of unfamiliar locations makes it essential to identify how their SA needs will be different from those of an on-site driver.

## SA demons for ROs

The difficulties of building and maintaining SA, often referred to as 'SA demons' have been widely documented in driving contexts, such as attention tunnelling, cognitive overload and out of the loop (OOTL) syndrome together with anxiety, fatigue and perception errors such as change blindness and errant mental models (Endsley 2012). However, there will be challenges in relation to OOTL syndrome, latency, embodiment, and workload that are specific to ROs of highly automated vehicles. We discuss each in turn in the next section and make suggestions as to how these SA risks can be mitigated.

### Out of the loop syndrome (OOTL)

SA is likely to develop differently for an RO compared with that of a driver who is present within the vehicle as they will be ‘out-of-the-loop’. OOTL is a major consequence of automation, leaving operators of automated systems handicapped in their ability to take over manual operations quickly in the event of automation failure (Endsley and Kiris 1995; Jones and Endsley 1996; Ottesen 2014; Porathe et al. 2014; Radlmayr et al. 2014). An RO in a remote call centre will be OOTL and it will take precious time for them to determine the cause of an edge case and successfully intervene (Endsley 2020). Attaining good SA in unknown remote environments is likely to be challenging for operators and they will experience a potentially hazardous delay while they build SA. Forming SA is also more challenging under time pressure, yet studies have found that operators will suspend all other tasks for up to 30% of their time to gain and re-gain sufficient SA, showing that maintaining SA is almost as difficult as attaining it (Drury et al., 2003; Larochelle et al. 2011, Porathe et al. 2014; Yanco and Drury 2004). An RO cannot begin to take control until they have built up adequate SA. This has serious implications if the location in which the AV has assumed the MRC presents a hazard for other road users, yet how much SA is 'enough' to start driving is hard to define even for a driver inside the vehicle.

Previous research into OOTL problems in highly automated driving have focused on the time taken to build up SA after take over requests (TORs) in Level 3 vehicles (for example Gold et al 2013, 2016; Lorenz et al. 2014; Melcher et al 2015; Radlmayr et al 2014; Salmon et al. 2012). Mok et al. (2017) found that 5–8 s are necessary to take back control of a Level 3 AV, after being engaged in an active secondary task (playing on a tablet). Eriksson and Stanton (2017) found that response times on average for on-road driving take over from Level 3 automation were around 3 s. However, as participants in Level 3 AVs are still inside the vehicle, sitting at the wheel, even with the distraction of the task it can be assumed that they still had some implicit SA which would not be available to an RO in a separate location (Gugerty 1997). An RO will be both cognitively and visually ‘blind’ prior to the TOR, so it is reasonable to assume there will be a longer delay for them to build up SA.

Likewise, in instances where the disengagement request from the AV is well-defined, for example 'sensor failure', ROs will be able to respond more quickly than if the AV cannot provide a cause, for example 'perception error' (California DMV 2019). In line with this assumption, Scholtz et al. (2004) found in field trials of semi-autonomous vehicles in multiple terrains that it took around 29 s for the remote operator to gain SA when it was specified that they needed to assist because of ‘Plan Failure’ (for example a plotted course did not succeed because the terrain was too bumpy). Yet, on average, it took 162 s to build up SA when the robot requested operator assistance but was not able to specify the cause of the problem. We can gauge from this that even when the RO knows why they have been asked to intervene, the time it takes to build up the necessary SA to take action is not trivial, but that far longer may be required in the event of an edge case where the RO has to work out the cause of the TOR.

### Latency issues for ROs

All autonomous driving is made possible through the use of mobile phone networks which transmit data (T Systems 2020). Assuming remote control over a self-driving car requires high amounts of data transfer and broad network coverage together with low latency (i.e. as small a delay as possible in the time it takes for a signal to be transmitted, in the case of an AV and teleoperated driving, from the car to an operator situated miles away). For an RO to be able to drive the vehicle in real time the time-lag between the signal and response of the car must be minimal otherwise turning, accelerating, and braking will all be delayed. Any latency of over 50 ms will mean that the image the RO is seeing is out of date in terms of useful interaction (T Systems 2020). This has led to debate in the industry as to whether remote management or teleoperated driving is even a genuine possibility as any latency will have detrimental safety effects on the operator's ability to build time-critical SA.

For remote operation to be viable, AVs must be able to guarantee consistent, real time streaming of all relevant data to the control centre to enable ROs to build and maintain SA. Proponents of teleoperated driving argue that there are already test cases that confirm latency is not an issue for RO SA on 4G networks. For example, Scania controlled a bus remotely using multiple camera feeds which reduce the need for high Megabits per second (Mbit/s), and Designated Driver teleoperated a car on the English coast from their offices in Portland, US (Ericsson 2017). However, the road environment frequently encounters obstacles to good network coverage such as tunnels, overhead trees and 9% of the UK, mainly in rural areas, are unable to get 4G coverage. This is a significant problem for remotely controlling AVs that require at least 50 Mbit/s to stream visual data from four HD cameras around the AV (OFCOM 2019).

Over 80 towns and cities across the UK now have 5G capability which has the potential to reduce latency to less than 10 ms (from 50 ms on 4G). Connected and autonomous vehicles can use 5G wireless services to send information between themselves and to remote call centres at faster speeds and with higher capacity, supporting RO's ability to quickly gain SA. 5G will also enable priority service provisioning to vehicles currently being teleoperated which will reduce the risk of network dropout and promises to pave the way for teleoperated driving, at any speed, to become part of AV business models for telecoms companies when adopted nationwide (Ericsson 2017; T Systems 2020). However, no matter how fast the transmission between AV and RO, restricting the sensory information that the RO is receiving to solely visual, the main type of information we have considered so far, will have an impact on their sense of embodiment in the vehicle. It may be critical to provide additional modes of sensory information to enhance the RO’s immersion in the remote scene when building SA.

### Embodiment issues and SA for ROs

Myriad human–robot disciplines have identified potential SA problems relating to missing sensory information (Ottesen 2014). Remote ship operators provide a good example in this respect. In rough seas, the autopilot is often disengaged and the ship steered by hand, enabling the handler to ‘feel’ the ship’s movement; this is not possible for the remote driver (Porathe et al. 2014, Jones and Endsley 1996). Similarly, when we manually drive a car, we feel ‘part’ of the vehicle even ducking instinctively as we go under a low bridge or ‘sucking in’ as we squeeze through a narrow space. An RO, not being physically present, is likely to miss this sense of *embodiment*; they cannot feel the seat beneath them or the pull of the wheel in their hands, they are in no personal danger and they are likely, until 5G is nationwide, to receive all visual and auditory information with at least some level of time-lag.

Even more concerning, limitations on ROs’ SA stemming from a lack of embodiment may result in a sense of detachment or reduced perception of risk (UNECE 2020). Remote operators have cited a sense of driving 'deaf' or feeling like it is a game, when the reality is that they are potentially driving real passengers with the resulting consequences if they crash only borne by those at the scene (Davies 2019). Even ROs offering remote assistance to passengers, without undertaking any remote driving, may lack the empathy or rapport that an on-board safety driver or conductor may share with fellow passengers (UNECE 2020). Although they may have access to a wider range of sensors from the AV system than if they were manually driving the car at the location, they have no vestibular feedback and so may misunderstand the conditions 'outside' or attribute greater significance to one piece of information than another (Endsley 2019). A lack of embodiment may also have a deleterious effect on speed perception; without force feedback pushing you back into the seat or information from the tyre friction on the road, it is difficult to accurately judge how fast you are driving and to remain engaged in the driving task (Tang et al. 2013). This may be addressed to some extent by remote operation training which teaches operators how to pick up cues from other feedback, for example spatial audio from microphones placed around the car, multiple viewpoints available at the same time, above, around and behind the car and enhancing video feeds with camera 'blur' to simulate speed cues or pop up with speed warnings (Störmer 2019; Tang et al. 2013; UNECE 2020).

### Workload issues for ROs

Unfortunately, one potential downside of providing this type of additional data is that the task of remote operation may then begin to push the boundaries of the “SA demon” of cognitive overload (Drury et al. 2007; Ottesen 2014, p. 2). Each additional piece of information provided carries a processing burden for ROs, who need to absorb the information and decide how to act. However, it is also theoretically possible that problems could arise due to a workload that is too low. For example, a situation in which the RO only has to deal with a low number of disengagements during a shift may exacerbate OOTL issues such as decreased vigilance. A careful consideration of the ways in which workload interacts with SA will therefore be essential in ensuring the safety of remote intervention. This would ideally disentangle the different types of workload which are assessed by standard measures such as the NASA Task Load Index (NASA-TLX), for example the mental, physical and temporal demands placed on the operator by the task and their resultant effects on performance, effort and frustration (Hart 2006).

Even the ways in which operators are allocated to jobs, including prioritising new AV requests when an operator is in the middle of a current call, will have important consequences for the operators’ workload (Daw et al. 2019). RO roles such as remote management, where ROs are in supervisory control, may allow easier division of attention across multiple assignments than direct teleoperation which requires total focus on a single vehicle (Cummings et al. 2020). Indeed, some remote management requests may be experienced simultaneously by many cars, for example in the event of an accident on the road, in which case they can be dealt with together with the same instruction, reducing workload. However, the workload could easily become too high under these conditions if too many vehicles are allocated, making the risk of errors more likely (UNECE 2020). Waymo is tackling this challenge by allocating separate support teams to different operations such as fleet technicians, fleet dispatch, fleet response and rider support which share the workload and allow for job specialisation (Waymo 2020).

Different forms of autonomy will also require more or less intervention than others and will therefore impose different levels of workload on ROs. Consider, for example, the difference between long distance autonomous trucking and local delivery robots. Although the robots operate at low speeds, perhaps implying a lower workload than high speed truck driving, teleoperating the ‘last mile’ local environment (as opposed to long distances on motorways) is more likely to involve busy or crowded situations, such as in a loading dock or fuel station, which creates higher demand on SA (UNECE 2020, StarskyRobotics 2020).

Carrying out remote operation work is likely to be stressful and highly specialised, thus there is an urgent need for training and regulation of ROs to ensure safe performance in this challenging and demanding role (Hampshire et al. 2020). Exploring technological developments which can make the interfaces used by ROs more intuitive, for example VR and head mounted displays (HMD) can reduce workload and improve RO SA (Hosseini and Lienkamp 2016). We discuss potential solutions to the challenges we have raised for operator SA in the final section.

## Suggested approaches for improving remote SA

In remote environments, it may be beneficial to make an RO’s driving experience as realistic as possible whereby visual, auditory and haptic cues are provided as if they were in-situ. An operator’s sense of embodiment relates both to their sense of location, feeling that they are occupying the same environment as the AV, and to their sense of body presence, feeling that they are physically inside the vehicle through sensory experience (Pamungkas and Ward 2014).

A naturalistic experience of driving even in remote contexts could be supplied by using a virtual display headset, allowing the operator to control their field of view just by moving their head (Almeida et al. 2014). This would avoid the need for multiple 2D monitors showing different camera feeds, which are likely to increase workload demand (Ricaud et al. 2017). Virtual Reality (VR) creates an illusion that the user is viewing the remote environment from an egocentric perspective, which seems likely to improve an ROs sense of embodiment in the scene. Their SA can also be enhanced by VR to provide a RO with a 360 view of the surrounding environment by combining LIDAR data with visual information from cameras and AR presented in a headset (Hosseini and Lienkamp 2016) Indicative of the potential success of applying VR to remote driving, Hyundai has released a prototype for a remote control system that allows an operator to drive a vehicle using binocular camera feeds to a VR headset that would give the vehicle operator a 3D view of the car’s immediate surroundings (United Lex 2017). If ROs are able to combine teleoperation with a sense of telepresence by using VR technology this should logically decrease the time it takes to build SA, by effectively narrowing the time–space detachment that has previously limited operators (Almeida et al. 2014). However, there are motion sickness issues with VR which develop as the vestibular input does not match the visual motion experience of the user (Hosseini & Lienkamp 2016). Current technological developments under design to address motion sickness include presenting a view of a fake steering wheel or the operator wearing VR gloves which show the driver's hands in the VR environment which aligns visual and motion cues (Manus 2020).

Augmented reality (AR) may facilitate better SA in ROs as it can provide extra information superimposed over the visual information provided via cameras to the RO, for example showing possible navigational paths that the operator could take (Brandão 2017). If the AV has had a failure on a busy motorway, AR could help the RO navigate the AV through three lanes of traffic safely by highlighting a safe route. Enhancing the video stream using AR can also improve operators’ depth perception and reduce workload as all the information is available in close proximity (Ruano et al. 2017). Placing AR virtual markers overlaid onto a map would allow ROs to ‘see’ salient information coming up which may be occluded by forward terrain or buildings (Ruano et al. 2017). NASA has tested a display system for cockpits that uses GPS to map an accurate picture of ground terrain creating an electronic picture that is unaffected by weather or time (NASA 2017). This would be invaluable when the remote environment is unfamiliar to the RO and the ‘drop in’ is unexpected and ambiguous, possibly under poor weather conditions. An RO, because of their remote location to the AV, would struggle to anticipate visual elements that are not directly apparent in the limited video feed. Augmented reality (AR) has been used to facilitate better SA by creating a “synthetic view” superimposed in the view of the RO using a heads-up display unit showing possible navigational paths that the operator could take that may be blocked from their direct view (Brandão 2017, p. 298).

Potentially, an RO of an AV that has unexpectedly requested assistance could request a short video summary that would show what happened seconds before to assist SA comprehension of the scene. 'Evocative summaries’ have been applied in multiple domains to present key visual information quickly and may provide helpful insights as to how best to present information to an RO (Russell and Dieberger 2002). Recent AV software company, AnyConnect, can give operators access to 4G/5G video, audio, and data recordings from a few minutes before the event, although it would depend on different use cases whether it would be more or less valuable to an RO to have a snapshot of what happened in comparison to images from the current scene.

Equally, ROs can gather more data from the environment around the remote AV if they have control over the cameras, which may be necessary in edge cases that are related to ambiguous information in the roadway. Giving ROs the autonomy to assess only parts of the environment that are of interest to them may reduce the exposure time it takes to build remote SA. However, the benefits of operator control are debatable, as humans are prone to errors. In the DARPA AAAI-2002 Robot Rescue Competition, one team's operator, during the first run of the arena, moved the robot’s camera off-centre to look at something more carefully. However, he then forgot to tap the screen to reposition the camera back to the centre, which meant he thought that the orientation of the camera was looking forward when it was actually 90° to the left. This SA error resulted in him driving the robot into the crowd (Yanco and Drury 2004).

Poor spatial and navigational awareness will reduce SA so providing ROs with pre-loaded terrain data with the AV’s current position superimposed on it, will give ROs better comprehension of 3D spatial relationships. However, driving is an inherently visuo-motor task so the video feed cannot be replaced with map tracking information solely. Although map-centric interfaces have been proven to be more effective in providing good location awareness, video-centric interfaces appear to improve awareness of surroundings so a combination of both on a graphical user interface would be ideal (Drury 2006). Many cars already have navigation systems as standard that show where the car has come from and their planned destination, so this information could be effectively transmitted to ROs to enable them to build stronger SA of the AVs location, spatially and navigationally (Yanco and Drury 2004). However, HD maps carry worrying safety failure rates as they may not update planned or unplanned road changes or unauthorised changes to road signs (Wood et al 2019).

It is important to reiterate that many of these suggestions for improving SA in ROs involve presenting additional information. Their benefits must therefore be carefully weighed against the additional workload that they will impose. We need to determine which is the most relevant information needed by ROs and how this can be transmitted to them efficiently, allowing them to build up SA quickly and effectively without risking cognitive overload. Future empirical research will be essential in exploring these issues to develop user interfaces that pull together concepts from research in other, related areas and applies them to the specific case of ROs of AVs. This work is currently underway in our laboratory.

## Data Availability

Not applicable to this review article.

## Abbreviations

AV: Autonomous vehicle(s)
SA: Situation awareness
RO: Remote operator
MRC: Minimal risk condition
ODD: Operational design domain
BSI: British Standards Institute
VR: Virtual reality
AR: Augmented reality
